# Supplementary material for: Effects Induced by the Temperature and Chemical Environment on the Fluorescence of Water-Soluble Gold Nanoparticles Functionalized with a Perylene-Derivative Dye
Source: Materials (Basel). 2024 Feb 28;17(5):1097. doi: 10.3390/ma17051097 (PMC10933863; doi:10.3390/ma17051097)
Supplement: Supplementary file 1 [file materials-17-01097-s001.zip › materials-2878199-supplementary.pdf]

Effects induced by the temperature and chemical environment on the fluorescence of water soluble gold nanoparticles functionalized with a perylene derivative dye.

Agnieszka Lindstaedt[1], Justyna Doroszuk[1], Aneta Machnikowska[1], Alicja Dziadosz[1], Piotr Barski[1], Vittoria Raffa[2], Dariusz Witt[1,3,*]

[1]ProChimia Surfaces Sp. z o.o., Sopot, 81-850, Poland

[2]Department of Biology, Università di Pisa, Pisa 56127, Italy

[3]Department of Organic Chemistry, Gdansk University of Technology, Gdansk, 80-233, Poland

*chemistry@prochimia.com, dariusz.witt@pg.edu.pl

UV-Vis measurements of PZPER and AuNPs functionalized with PZPER at variable temperature

When the UV-Vis spectra were recorded for aqueous solution of PZPER at variable temperature, then the minor changes at 540 nm could be observed (Figure S1).

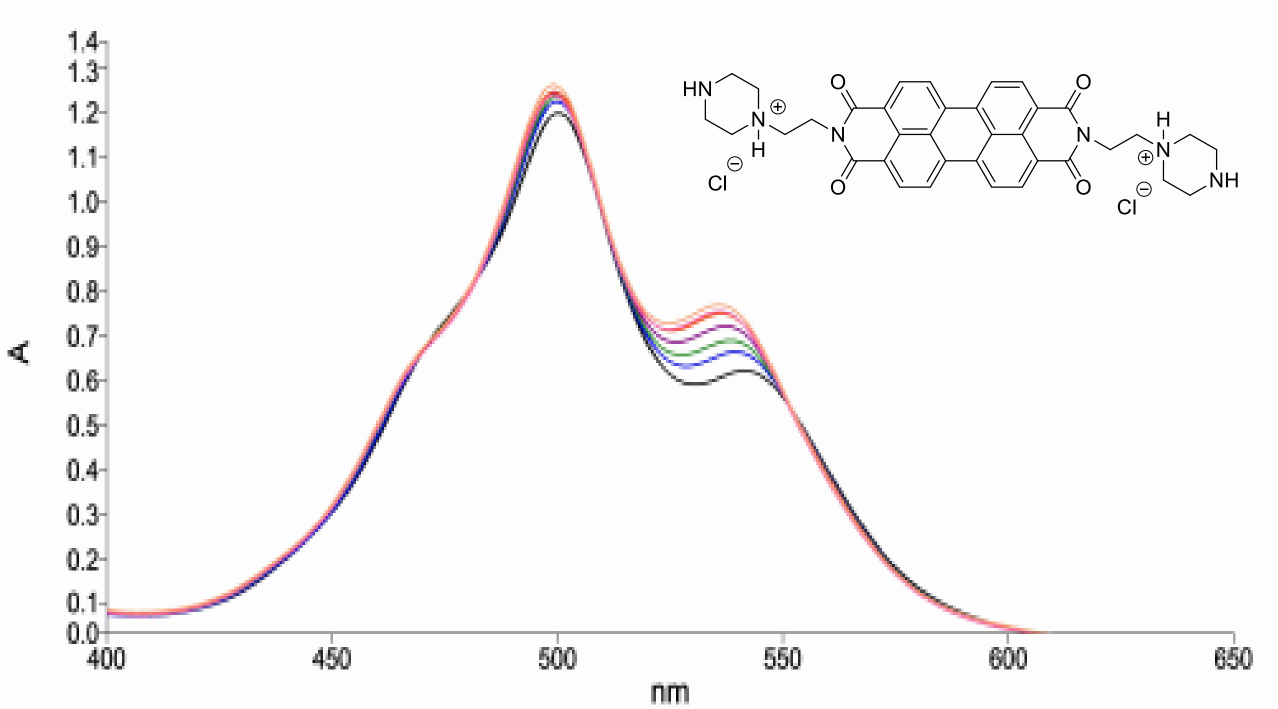

Figure S1. UV–Vis spectra of PZPER (50 μM) in water at 25, 50, 60, 70, 75, 80 and 85 °C.

UV-Vis spectra of PZPER in water showed a λ_{max} value centered at 500 nm. According to literature, the observed changes at 540 nm can be related to the formation of H-aggregates of dyes due to the π–π stacking of chromophores in a ladder fashion, also at very low dye concentration.

Hoverer, UV-Vis measurement of AuNPs functionalized with PZPER (5 μM) provided only minor changes in recorded spectra. Probably, the amount of chromophore is insufficient to observe a reliable response vs. temperature (Figure S2).

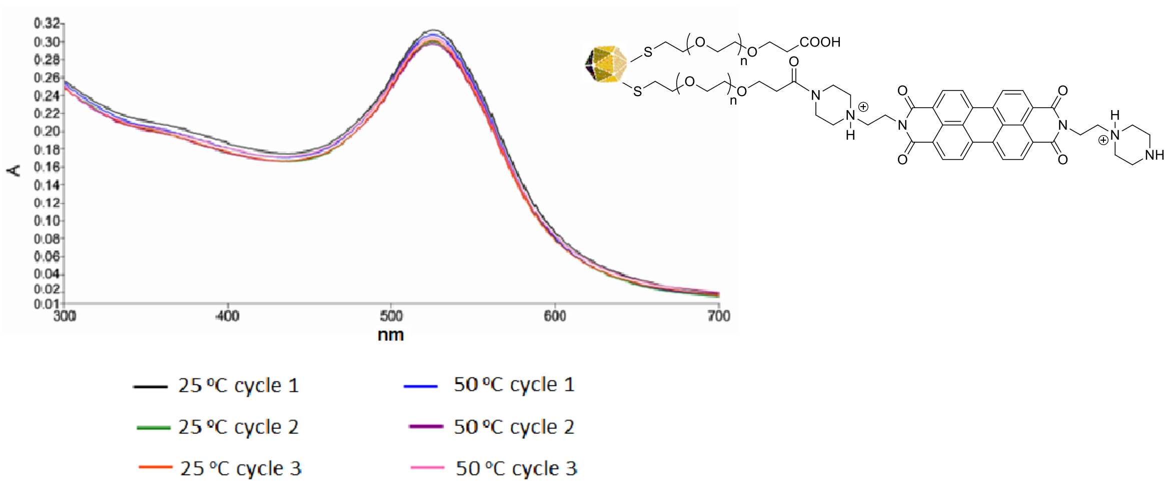

Figure S2. UV–Vis spectra of AuNPs functionalized with PZPER (5 µM) in MOPS buffer (1 mM, pH 5.35) in 3 cycles 25 and 50 °C.

We performed 3 cycles, with three measurements at 25 and 50 °C. Although the changes of UV-Vis spectra can not be related to variable temperature, the performed cycles proved the thermal stability of functionalized AuNPs.

Determination of PZPER amount attached to AuNPs

The solution of AuNPs was treated at pH 1 with HCl solution for 30 min. then precipitated gold was separated and solution was taken for fluorescence measurement. Based on the calibration curve at pH 1 the concentration of PZPER released from AuNPs was 5 µM, what corresponds to 16% of all carboxylic groups functionalized with PZPER. The sample from decomposed AuNPs with released PZPER was diluted 128 times and observed fluorescence was 2870. The linear calibration curve (Figure S3) C = (FL + 252.39)/78957, when FL=2870 then C = 0.039545, after multiplying 128 the concentration of released PZPER is 5.06 µM. The original released PZPER solution was diluted form 32 to 256 times but after recalculation the final concentration of PZPER was always 5.06 µM.

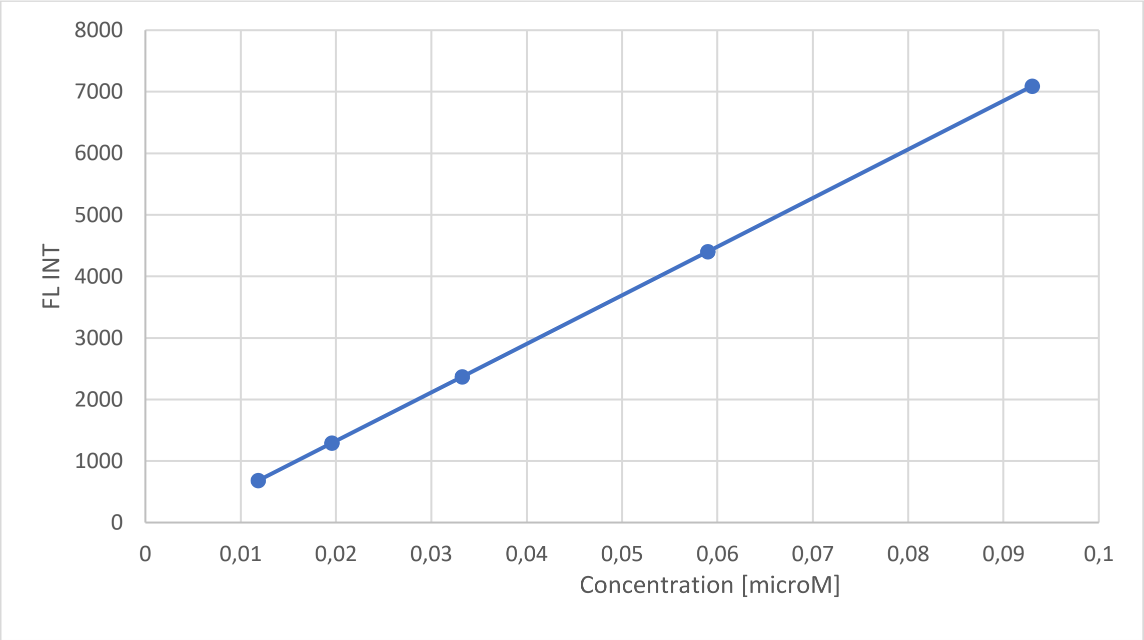

Figure S3. The linear calibration curve of PZPER at pH 1

AuNRs Synthesis

BDAC/CTAB ratio 0.43; Ag/Au ratio 0.14

The synthesis of gold seeds stabilized with CTAB

$HAuCl_4$ + CTAB + $NaBH_4$ → gold seeds CTAB
(1) (2) (3)

	Reagent	M [g/mol]	C [mol/dm^3]	n[mmol]	m[mg]	V[mL]
1	$HAuCl_4$	339,79	0,0005	0,0025	0,85	5
2	CTAB	364,45	0,2	1	364,45	5
3	$NaBH_4$	37,83	0,01	0,006	0,23	0,6

All reagents should be warmed up to RT. The synthesis is carried out in an incubator set at 33°C to avoid crystallization of the CTAB. Cold water for dissolving $NaBH_4$ should be prepared.

A falcon tube was charged with CTAB (1 mmol, 364.45 mg) (Sigma Aldrich; cat# 52365-50G) and degassed, deionized water (5 mL). CTAB was dissolved it in a water bath at 40°C (CTAB is not dissolved in water at RT). A solution of $HAuCl_4$ (2.5 µmol, 0.85 mg) (Acros Organics; cat# 437170010) was prepared in a falcon tube in deionized, degassed water (5 mL). Then a solution of $HAuCl_4$ and CTAB was poured into a conical flask with stirring. A solution of $NaBH_4$ (6 µmol, 0.23 mg) (Sigma Aldrich; cat# 71320-25G) was prepared in cold, deionized, degassed water (0.6 mL) and was added to a stirred mixture of $HAuCl_4$ and CTAB. The reaction mixture turns from yellow to light brown. The obtained seeds solution was immediately used for preparation of AuNRs.

The synthesis of AuNRs stabilized with CTAB and BDAC

$HAuCl_4$ + BDAC + CTAB + $AgNO_3$ + Ascorbic Acid + seeds CTAB → AuNRs
(1) (2) (3) (4) (5) (6)

	Reagent	M [g/mol]	C [mol/dm^3]	n[µmol]	m[mg]	V[ml]
1	$HAuCl_4$	339,79	0,001	5,0	1,7	5
2	BDAC	396,09	-	300	118,8	5
3	CTAB	364,45	-	700	255,11	-
4	$AgNO_3$	169,87	0,004	0,7	0,12	0,17
5	Ascorbic Acid	176,12	0,0778	5,45	0,96	0,07
6	Seed solution	-	-	-	-	0,1

All reagents should be warmed up to RT. The synthesis is carried out in an incubator set at 33°C to avoid crystallization of the CTAB.

The following solutions were prepared in deionized, degassed water:

1. $HAuCl_4$ (Acros Organics; cat# 437170010) (5.0 µmol, 1.7 mg) in water (5 mL).
2. BDAC (Sigma Aldrich; cat# B4136-100G) (300 µmol, 118.8 mg) and CTAB (Sigma Aldrich; cat# 52365-50G) (700 µmol, 255.11 mg) in water (5 mL) at 40°C (BDAC/CTAB ratio 0.43).
3. $AgNO_3$ (Sigma Aldrich; cat# 209139-25G) (0.7 µmol, 0.12 mg) in water (0.17 mL)
4. L-Ascorbic Acid (Sigma Aldrich; cat# A92902-25G) (5.45 µmol, 0.96 mg) in water (0.07 mL)

A solution of $HAuCl_4$ (5m L) and BDAC/CTAB (5 mL) was added to a conical flask with stirring. Next, a solution of $AgNO_3$ (0.17 mL) and L-Ascorbic acid (70 μL) was added. A solution becomes colorless. Then a fresh Seeds-CTAB solution (0.1 mL) was added. The color changed from violet to red. After 15 min. the solution was centrifuged (30min, 9600rpm). The supernatant was discarded and the residue was dissolved in distilled, degassed water (0.5 mL). The synthesis was performed in six parallel conical flasks. The solutions were combined and UV-VIS (Figure S4) and TEM (Figure S4) analysis was performed. Concentration of AuNRs 16.9 10^{14} (NR/ mL), with average size 8.8 x 39.8 nm

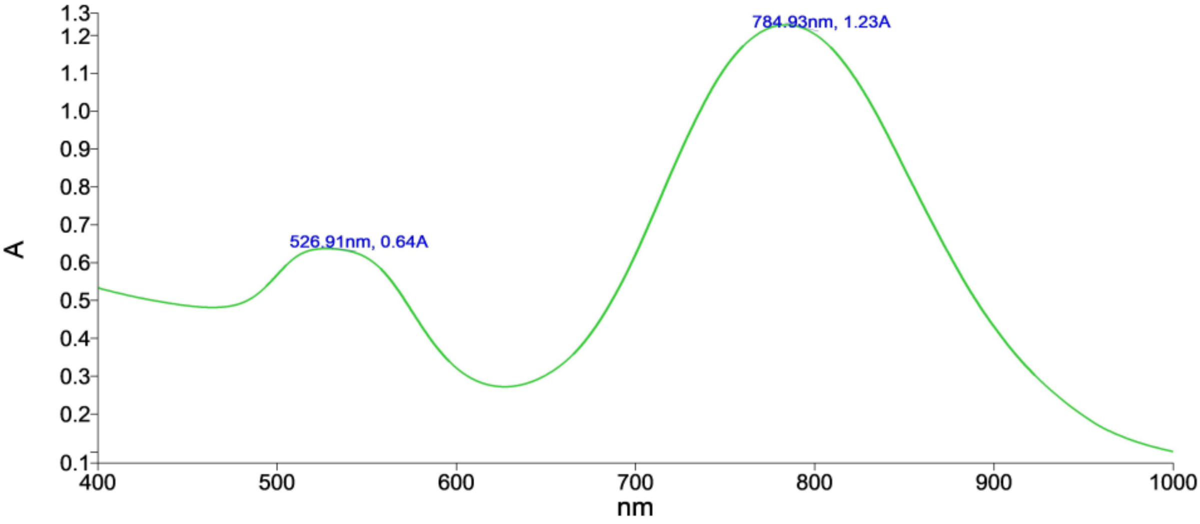

Figure S4. UV-Vis spectrum of AuNRs stabilized with CTAB in water.

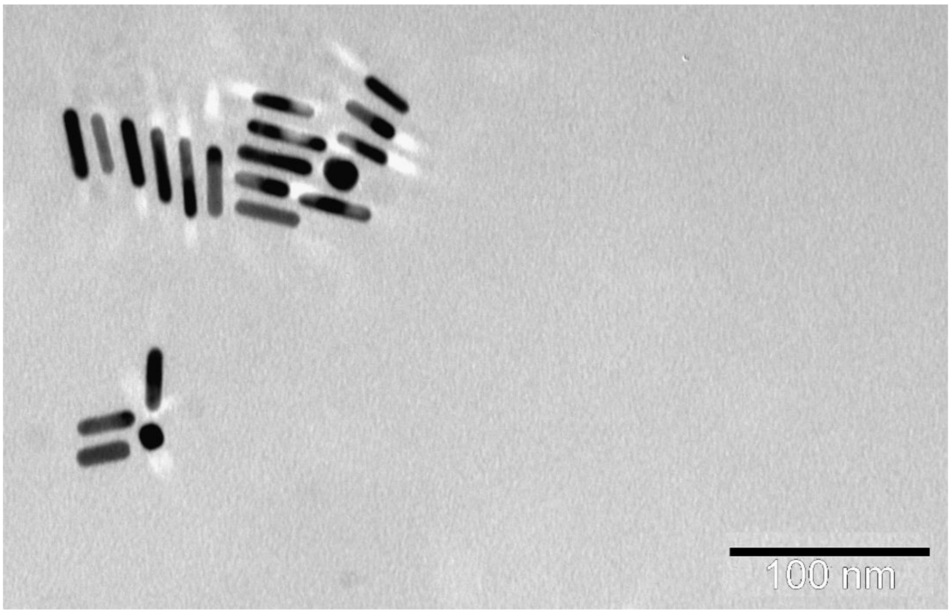

Figure S5. TEM image of AuNRs stabilized with CTAB/BDAC.

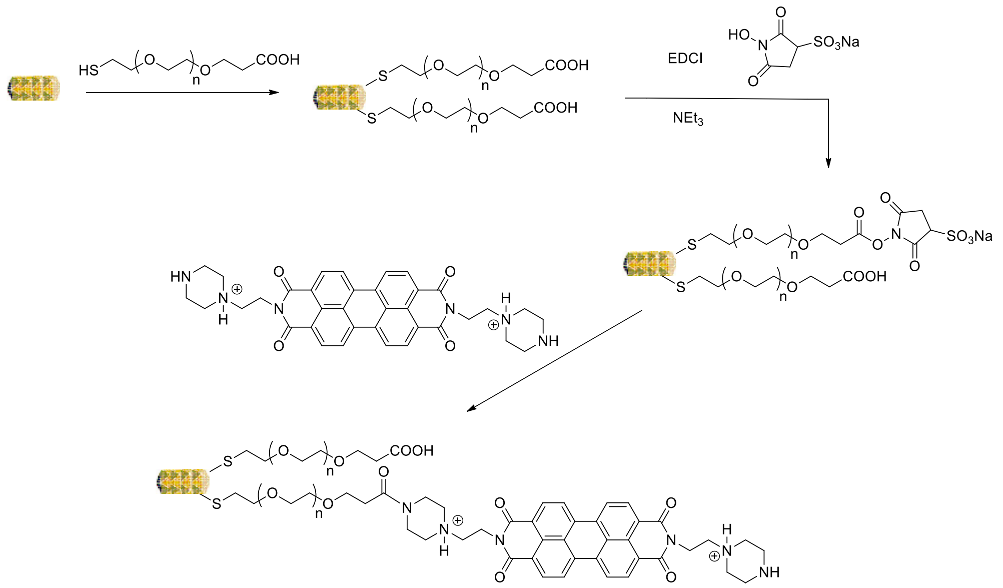

Figure S6. Synthesis of AuNRs functionalized with PZPER.

Synthesis of AuNR_PEG3000_COOH (Figure S6)

A solution of HSPEG3000COOH (10 µmol) (IRIS Biotech; cat# PEG1099) in deionized, degassed water (1 mL) was added to a solution of AuNRs BDAC/CTAB (10 µmol, 10 mL). The solution was stirred at RT for 1h and then at 33°C overnight. The excess of reagent was removed by dialysis against water (3 times, MWCO 3,5kDa). UV-VIS and TEM (Figure S7) analysis was performed.

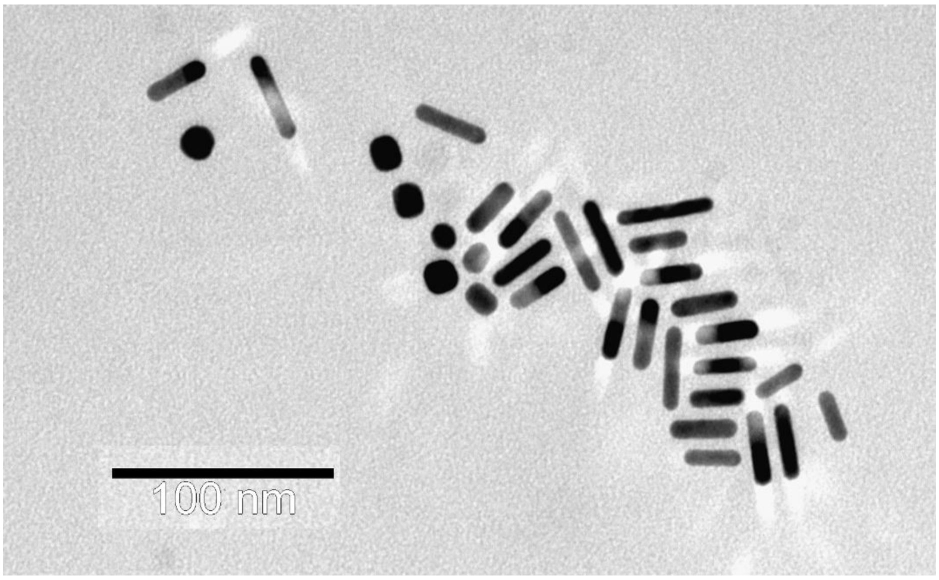

Figure S7. TEM image of AuNRs stabilized with HS-PEG3000-COOH.

Synthesis of AuNR_PEG$_{3000}$_PZPER (Figure S6)

Triethylamine (2 eq. 10 µmol) (Sigma-Aldrich ; cat# 8.08352) in 1 mL of deionized, degassed water was added to solution of AuNRs_PEG$_{3000}$COOH (5 µmol in 10 mL of water) and leave with stirring for 5min. Next, a solution of EDCI (4 eq. 20 µmol) (Sigma-Aldrich; cat.# 8.00907) in 1 mL of deionized, degassed water was added to the mixture and stirred for 10 min. Then, a solution of *N*-hydroxysulfosuccinimide

sodium salt (4 eq. 20 µmol) (Sigma-Aldrich; cat.# 56485) in 1 mL of deionized, degassed water was added to the mixture and stirred for 25 min. Finally, a solution of PZPER hydrochloride (2 eq. 10 µmol) in 1 mL of deionized, degassed water was added to the mixture and stirred for 1h. The excess of reagent was removed by centrifugation with Amicon filter (MWCO 50kDa; 9600 rpm; 20 min) and washed twice with deionized water. UV-VIS (Figure S8) analysis was performed. Concentration of AuNRs 16.9 10^{14} (NR/ mL), with average size 8.8 x 39.8 nm (Figure S9).

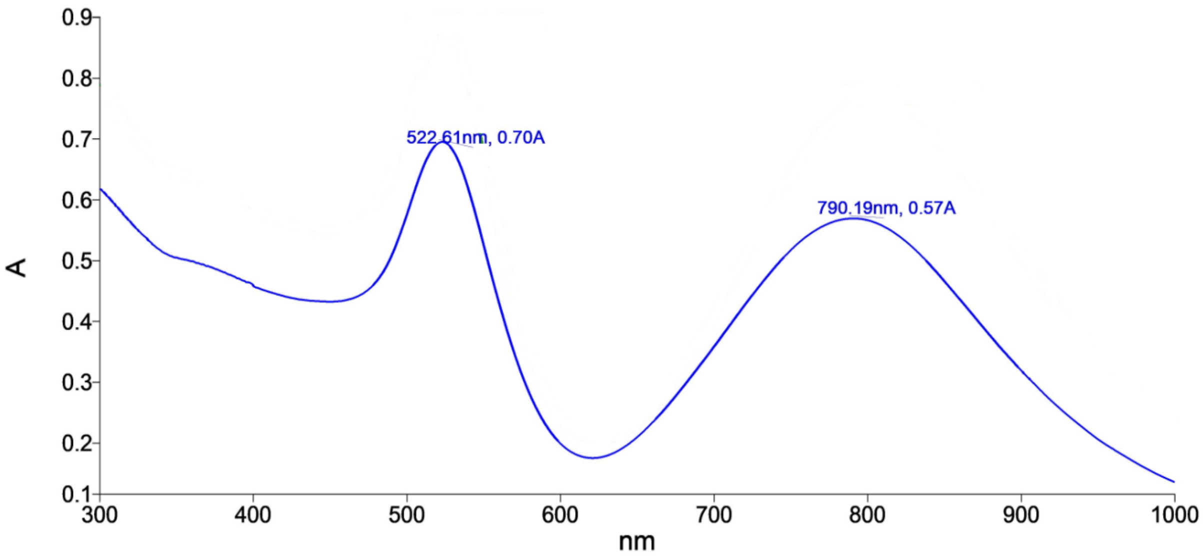

Figure S8. UV-Vis spectrum of AuNRs (13.4 10^{14} NR/mL) functionalized with PZPER in water.

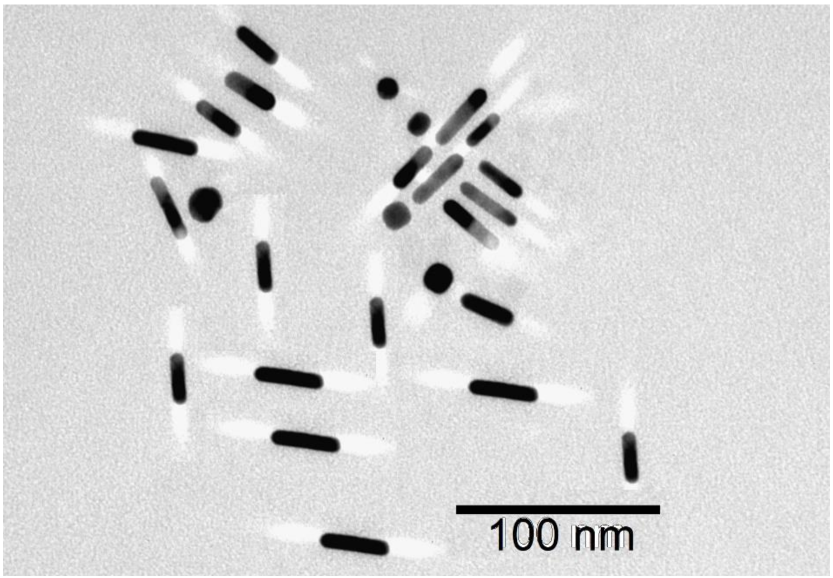

Figure S9. TEM image of AuNRs functionalized with PZPER.

Determination of PZPER amount attached to AuNRs

The solution of AuNRs was treated at pH 1 with HCl solution for 30 min. then precipitated gold was separated and solution was taken for fluorescence measurement. Based on the calibration curve at pH 1 the concentration of PZPER released from AuNRs was 0.8 µM. The sample from decomposed AuNRs

with released PZPER was diluted 8 times and observed fluorescence was 8186. The linear calibration curve (Figure S10) C = (FL + 252.39)/78957, when FL=8186 then C = 0.106873, after multiplying 8 the concentration of released PZPER is 0.85 μM. The original released PZPER solution was diluted from 4 to 32 times but after recalculation the final concentration of PZPER was always 0.85 μM.

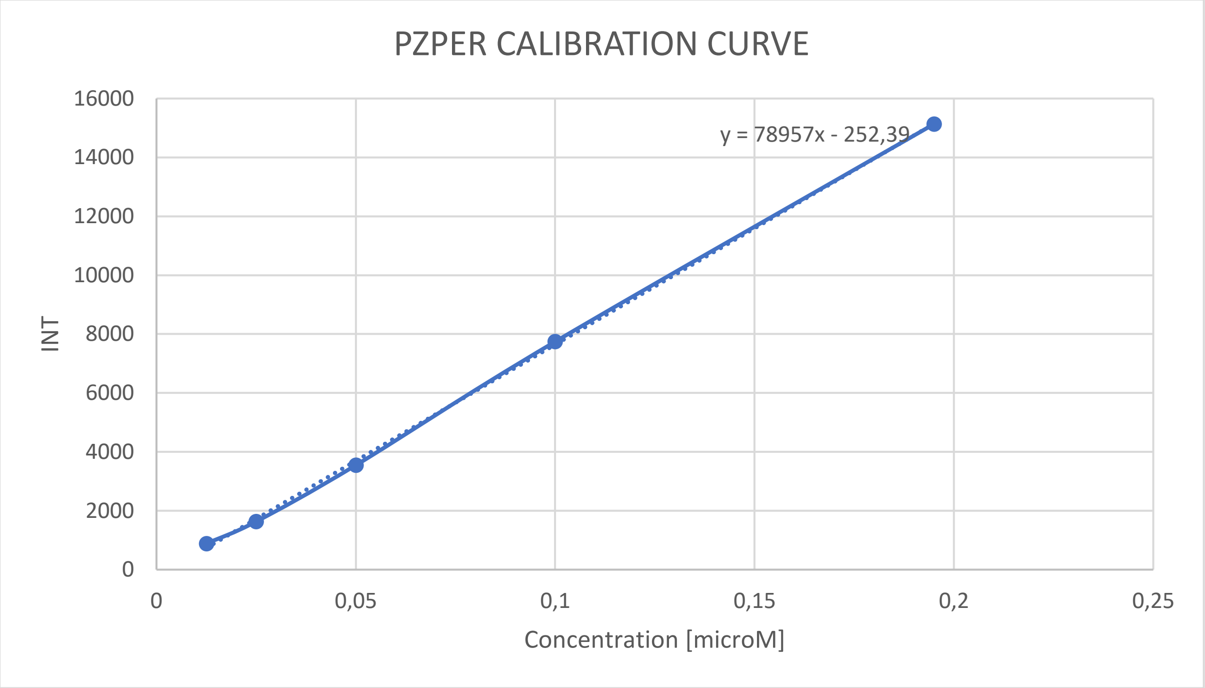

Figure S10. The linear calibration curve of PZPER at pH 1

UV-Vis measurements of AuNRs functionalized with PZPER.

When the presence of PZPER for AuNRs was confirmed (0.85 μM), the UV-Vis measurement for AuNRs_PEG3000_COOH (without PZPER) (blue), AuNRs_PEG3000_PZPER (functionalized with PZPER 0.85 μM) (red) and PZPER (0.85 μM) (green) were compared (Figure S11).

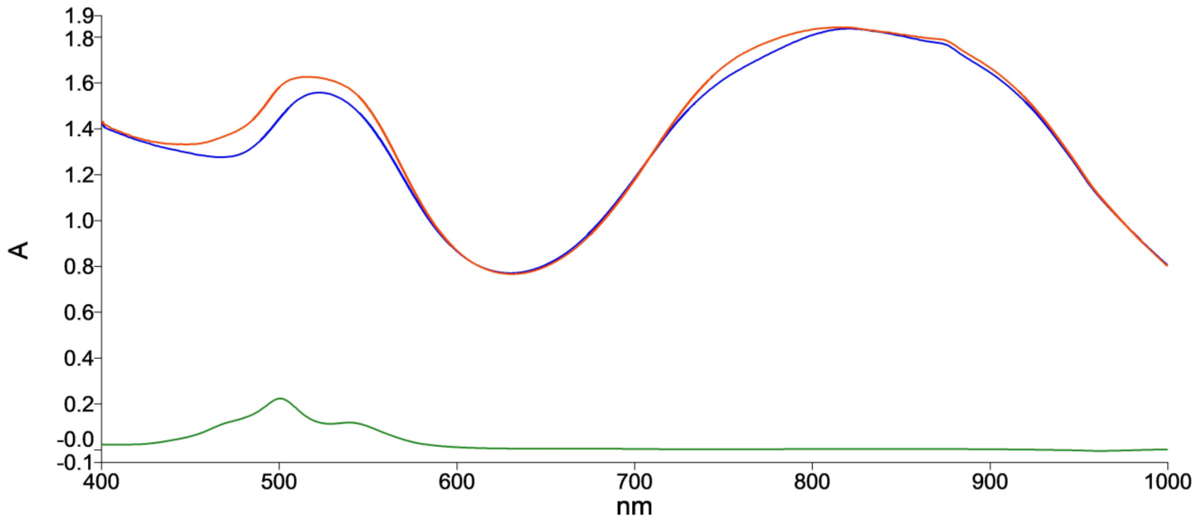

Figure S11. UV-Vis AuNRs_PEG3000_COOH (without PZPER) (blue), AuNRs_PEG3000_PZPER (functionalized with PZPER 0.85 μM) (red) and PZPER (0.85 μM) (green)

As can be seen for AuNRs the presence of attached PZPER cannot be confirmed in reliable way by UV-Vis (Figure S11). The amount of attached PZPER (0.85 μM) is relatively small and the signal is overlapping with plasmon of nanorods at ca 520 nm.

Fluorescence of AuNRs functionalized with PZPER.

We performed fluorescence measurement of AuNRs functionalized with PZPER (Figure S12). However, the observed fluorescence was very low. The observed low fluorescence probably resulted from relatively low PZPER concentration (0.85 μM) and overlapping of excitation wavelength (λ_{ex} = 532 nm) with AuNRs plasmon (524 nm).

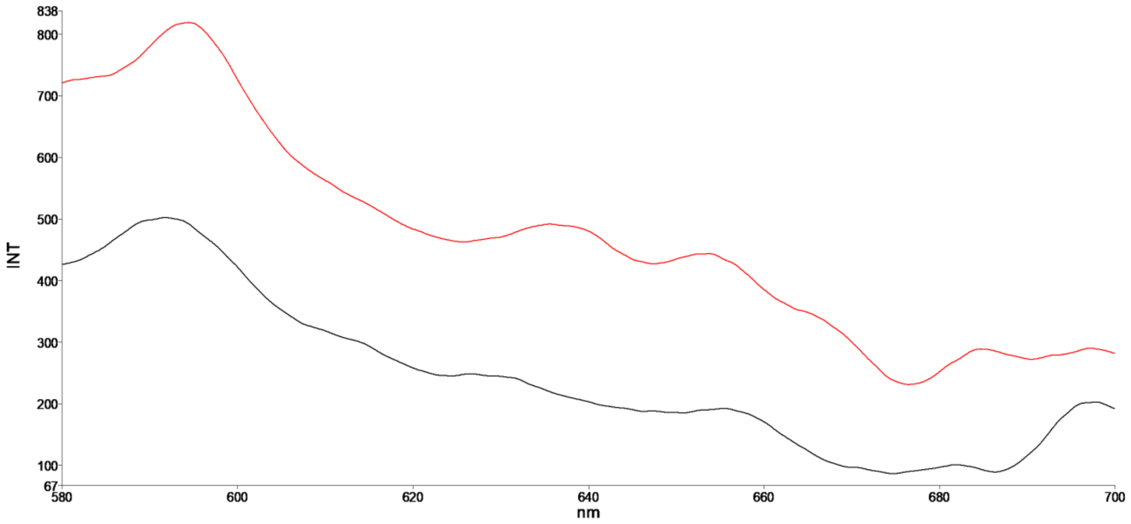

Figure S12. Fluorescence spectra of AuNRs (13.4 x10^{14} NR/mL black and 16.9 x10^{14} NR/mL red) functionalized with PZPER (λ_{ex} = 450 nm) in MOPS buffer (1 mM, pH 5.35) at room temperature.

When measurement was repeated at variable temperature then correlation of fluorescence with temperature could not be confirmed in reliable way (Figure S13).

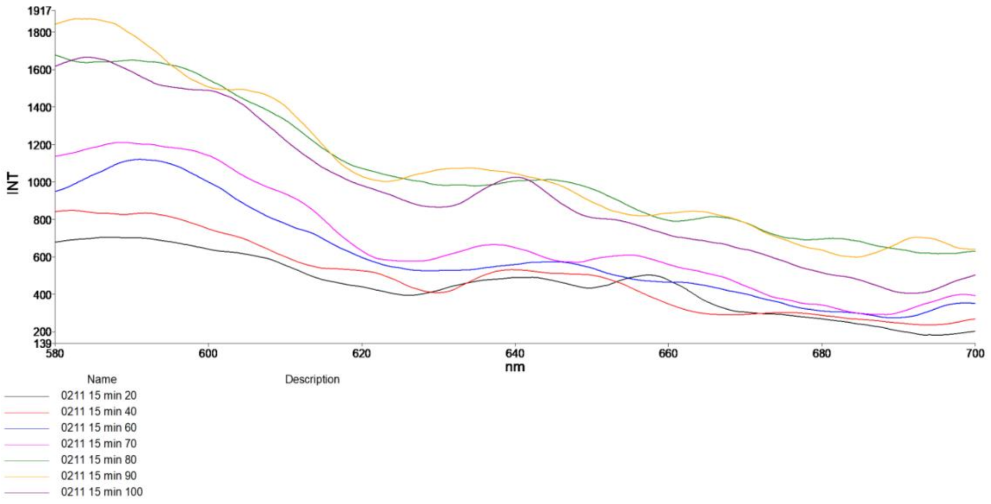

Figure S13. Fluorescence spectra of AuNRs (16.9x10^{14} NR/mL) functionalized with PZPER (λ_{ex} = 450 nm) in MOPS buffer (1 mM, pH 5.35) at variable temperature.

The intensity of fluorescence at 590 nm is not changed in linear way vs temperature and for 80, 90 and 100 °C the order is disturbed.
